# Molybdopterin biosynthesis pathway contributes to the regulation of SaeRS two-component system by ClpP in *Staphylococcus aureus*

**DOI:** 10.1080/21505594.2022.2065961

**Published:** 2022-04-28

**Authors:** Na Zhao, Yanan Wang, Junlan Liu, Ziyu Yang, Ying Jian, Hua Wang, Mahmoud Ahmed, Min Li, Taeok Bae, Qian Liu

**Affiliations:** a Department of Laboratory Medicine, Ren Ji Hospital, School of Medicine, Shanghai Jiao Tong University, Shanghai, China; b Department of Biology, Indiana University Northwest, Gary, IN, USA; c Department of Microbiology and Immunology, Indiana University School of Medicine-Northwest, Gary, IN, USA

**Keywords:** *Staphylococcus aureus*, proteolysis system, SaeS, MoeA, degradation

## Abstract

In *Staphylococcus aureus*, the SaeRS two-component system is essential for the bacterium’s hemolytic activity and virulence. The Newman strain of *S. aureus* contains a variant of SaeS sensor kinase, SaeS L18P. Previously, we showed that, in the strain Newman, SaeS L18P is degraded by the membrane-bound protease FtsH. Intriguingly, the knockout mutation of *clpP*, encoding the cytoplasmic protease ClpP, greatly reduces the expression of SaeS L18P. Here, we report that, in the strain Newman, the positive regulatory role of ClpP on the SaeS L18P expression is due to its destabilizing effect on FtsH and degradation of MoeA, a molybdopterin biosynthesis protein. Although the transcription of *ftsH* was not affected by ClpP, the expression level of FtsH was increased in the *clpP* mutant. The destabilizing effect appears to be indirect because ClpXP did not directly degrade FtsH in an *in vitro* assay. Through transposon mutagenesis, we found out that the *moeA* gene, encoding the molybdopterin biosynthesis protein A, suppresses the hemolytic activity of *S. aureus* along with the transcription and expression of SaeS L18P. In a proteolysis assay, ClpXP directly degraded MoeA, demonstrating that MoeA is a substrate of the protease. In a murine bloodstream infection model, the *moeA* mutant displayed reduced virulence and lower survival compared with the WT strain. Based on these results, we concluded that ClpP positively controls the expression of SaeS L18P in an FtsH and MoeA-dependent manner, and the physiological role of MoeA outweighs its suppressive effect on the SaeRS TCS during infection.

## Introduction

*Staphylococcus aureus* is an important human pathogen causing a broad range of diseases from minor skin infections to life-threatening infections, such as pneumonia, bacteremia, and osteomyelitis [1]. The bacterium can evade host immune response by producing various toxins, cell surface molecules, and immune-modulating proteins [2]. The production of these virulence factors is controlled by a series of regulatory systems, including the SaeRS two-component system (TCS) [3].

The SaeRS TCS, consisting of the sensor kinase SaeS and the response regulator SaeR, can be activated by cognate signals such as human neutrophil peptides [4]. Upon stimulation, SaeS autophosphorylates its histidine residue; then, the phosphoryl group is transferred to the aspartic residue of SaeR. The phosphorylation enables SaeR to bind its target genes and modulates the transcription of the targets (e.g. alpha-hemolysin [*hla*] and nuclease [*nuc*]) [5,6]. Several variants of SaeS have been reported in clinical strains. Importantly, the polymorphisms of SaeS affect the Sae activity. SaeS^SKT^, with three substitution mutations at N227S, E268K, and S351T in ST30 and ST36 lineages of *S. aureus*, displayed the altered activation pattern of the SaeRS system [7]. In the Newman strain, the SaeS protein is constitutively active due to the L18P mutation in the first transmembrane helix [8,9]. Intriguingly, the L18P mutation not only increased the autokinase activity of SaeS but also rendered the protein susceptible to proteolysis by FtsH, a membrane-bound ATP-dependent protease [10].

ATP-dependent proteolysis systems maintain protein homeostasis by degrading unfolded or misfolded substrate proteins [11]. In *S. aureus*, there are three ATP-dependent proteases:ClpP, HslV (ClpQ), and FtsH [12]. The ClpP protease system utilizes the ATPase partners ClpB, ClpC, ClpL, and ClpX, which exhibit chaperone-like functions [13]. The ClpP proteolytic activity is required for virulence, stress response, and physiological homeostasis in *S. aureus* [14], whereas HslUV plays a minor role in stress survival [15]. FtsH is an ATP-dependent protease located in the membrane. In *S. aureus*, it is required for stress resistance and virulence [16]. In particular, in the Newman strain, FtsH controls the SaeRS TCS by degrading the L18P variant of SaeS [10]. Here, we found that, in the strain Newman, the expression level of SaeS L18P is also positively controlled by ClpP, a protease in the cytoplasm. We further identified that the positive role of ClpP is due to its destabilizing effect on FtsH and degradation of MoeA, a cytoplasmic enzyme involved in the synthesis of the molybdenum cofactor.

## Methods

### Ethics statement

Human heparinized venous blood was obtained from healthy volunteers following a protocol approved by the ethics committee of Renji Hospital, School of Medicine, Shanghai Jiao Tong University, Shanghai, China. The written informed consent were obtained from all individuals before donating blood.

The animal experiment was approved by the ethics committee of Renji Hospital, School of Medicine, Shanghai Jiaotong University. We try our best to minimize the suffering of the animals.

### Bacterial strains and growth conditions

The *S. aureus* strains and plasmids used in this study are listed in Supplementary Table S1. Various media including lysogeny broth (LB) and tryptic soy broth (TSB) was used for *Escherichia coli* and *S. aureus* culture, respectively. However, heart infusion broth (HIB) supplemented with 5 mM CaCl_2_ was used for transduction of plasmids. When necessary, antibiotics were added to the growth media at the following concentrations: ampicillin, 100 μg/ml; erythromycin, 10 μg/ml; kanamycin, 50–100 μg/ml and chloramphenicol, 10 μg/ml.

### Reagents

The restriction enzymes and DNA modification enzymes (New England Biolabs) were used for plasmid construction. The plasmid miniprep kit (Omega) was used for plasmid extraction according to the manufacturer’s instructions. Plasmid DNA was transformed *E. coli* by the method of Hanahan and Meselson [17] and electroporated into *S. aureus* RN4220 with a gene pulser (Bio-Rad). The plasmids from RN4220 were introduced into target strains of *S. aureus* by transduction with ϕ 85.

### Construction of plasmids for gene knockout and complementation

A ligation independent cloning (LIC) method was used for *clpQ* deletion [18]. Briefly, both the pIMAY vector and the flanking fragments of *clpQ* were amplified by PCR using PrimSTAR (Takara). The primers used for the amplification were listed in Supplementary Table S2. After treatment with T4 DNA polymerase in the presence of dGTP (vector) or dCTP (insert DNA), the PCR products were mixed and transformed *E. coli* DH5α. The resulting plasmid pIMAYΔ*clpQ* was introduced into the strain Newman as mentioned above. The deletion was acquired according to the protocol mentioned in the literature [19].

The plasmid pKOR1 was used for the deletion of the *moeA* gene. The plasmid was constructed and introduced into the strains Newman using the same method as above. The deletion was carried out according to the protocol for pKOR1 [20].

To construct *moeA* complement plasmid, *moeA* was PCR-amplified using the primers PN5/6 (Supplementary Table S2). The restriction enzyme *Sma*Ι/*Bam*HΙ were used for the pOS1 and amplified fragment digest [21]. The resulting plasmids pOS1-*moeA* were introduced into the strain NM∆*moeA* for the complementation test. To express MoeA proteins *in vitro*, *moeA* was amplified using the primers PN7/8 by PCR. The enzymes *Eco*RI/*Bam*HI were used for pET28a-*moeA* construction. The protein was expressed in the strain *E. coli* BL21 (DE3).

### Real-time quantitative reverse transcription-PCR (RT-PCR)

The bacteria was cultured in 3 ml TSB in tubes (15 ml) with shaking (200 rpm) at 37°C and harvested at stationary phase (OD_600_ ≈2). The Mini-Beadbeater (Biospec Products) was used for cell disruption at maximum speed for 30 s. After incubation on ice for 5 min, the suspension was centrifuged. The supernatant was used for RNA extraction according to the manufacturer’s instructions (Qiagen). Totally, 1 μg of total RNA was used for DNA removal using a TURBO DNA-freeTM kit (Ambion). The HieffTM first Strand cDNA Synthesis Super Mix for RT-PCR kit (Yeasen Bio, China) was used for cDNA synthesis. The following real-time PCR was performed by SYBR-Green PCR reagents (Roche) using cDNA as a template on a 7500 Sequence Detector (Applied Biosystems). Primers used for RT-PCR are listed in Supplementary Table S2. All RT-PCR experiments were performed using *gyrB* as an internal control.

### Western blot hybridization

The test strains were diluted 1:100 into fresh TSB (3 ml) in tubes (15 ml) from overnight culture. After shaking at 37°C (200 rpm) for 8 h, the bacteria were collected and normalized to OD_600_ = 2. The cell pellets were treated with lysostaphin (50 μg/ml) for 30 min at 37°C and mixed with 2 × SDS loading buffer. Western blot analysis was carried out by following the protocol described in the literature [22]. The FtsH and MbtS antibody was produced by GLbiochem, China. The Hla and nuclease antibodies were purchased from Abcam and USBiological, respectively. The His-Ab was purchased from Sigma. All other antibodies were produced by GenScript.

### Nuclease activity assay

We collected the bacterial culture supernatants by passing through a 0.22 μm filter. After dilution with fresh TSB (1:10), the cell-free supernatants were incubated with 500 μg/ml salmon sperm DNA (Sigma) at 37°C At the different time points, samples were mixed with 6× loading buffer. 1% agarose gel with ethidium bromide was used for DNA visualization.

### Protease assay *in* *vitro*

The His-ClpX, His-ClpP and His-MoeA proteins were purified as described by Ni [23]. Briefly, the *E. coli* BL21 (DE3) strain carrying the corresponding plasmid was grown in LB with 50 μg/ml kanamycin to exponential growth phase (OD_600_ = 0.6). then we added 0.5 mM isopropyl-beta-D-thiogalactopyranoside (IPTG) for inducing protein expression. The culture was further incubated at 16°C overnight. The His-ClpX, His-ClpP, and His-MoeA fusion proteins were purified with Ni-NTA agarose (Novagen) according to the manufacturer’s instruction. Proteins were eluted in lysis buffer (10 mM Tris, pH 7.4, 500 mM NaCl, 5% Glycerol, pH 7.4) containing 500 mM imidazole. Purified proteins were concentrated and dialyzed against the dialysis buffer (10 mM Tris-Cl, pH 7.5, 15% (w/v) glycerol, 50 mM KCl, 5 mM MgCl_2_), using a 10 K molecular weight dialysis cassette (Sangon Co.). The Bradford Protein Assay kit (Sangon Co.) was used for the qualification of protein concentration using bovine serum albumin as a standard.

For protease degradation assay, 3 μM ClpX, 5 μM ClpP, 8 mM ATP, and an ATP regeneration system (80 μg/mL creatine kinase and 40 mM creatine phosphate) were mixed within reaction buffer containing 50 mM HEPES-KOH (pH 7.6), 50 mM KCl, 20 mM MgCl_2_, 1 mM EDTA, 5% (w/v) glycerol, and 1 mM DTT. The assay was performed at 37°C when 6 μM purified His-FtsHc, His-MoeAor 0.5 mg/ml α-Casein (Sigma C6780) was added to the mixture. At each given time point, 15 μl sample taken from the assay was mixed with 2 × SDS loading buffer. The samples were subjected to SDS-PAGE and the protein was visualized by coomassie blue staining.

### Transposon mutagenesis of *S. aureus* Newman

Transposon mutagenesis was performed using plasmid pMA15. The plasmid has a chloramphenicol (chl)-resistance marker and can deliver a mariner transposon with kanamycin (kana) resistance marker. The plasmid also contains an improved temperature-sensitive replicon repC3 of pT181, and its replication is blocked at 42°C [24]. First, pMA15 was transduced with ϕ85 into a *clpP* transposon mutant of Newman strain, where the transposon in *clpP* confers resistance to erythromycin.The transductant was inoculated into 10 ml fresh TSB containing 100 μg/ml kana and 10 μg/ml chl. After shaking overnight at 30°C (200 rpm), the culture was diluted at 1:100 into 10 ml fresh TSB containing 100 μg/ml kana and cultured overnight at 42°C with shaking. This process was repeated twice.The putative transposon insertion mutants (kan^R^ and chl^S^) were selected and were used for library screening.

### Lysis of erythrocytes by culture filtrates

After culture for 18 h, the culture filtrates were collected and incubated with human red blood cells (2% v/v in PBS) in a 96-well round-bottom plate. After incubation at 37°C for 1 h, the plate was centrifuged at 4000 rpm for 10 min, and the supernatant was transferred into another 96-well flat-bottom plate. The optical density at 540 nm was measured on a plate reader (Synergy; BioTek). The assay was performed in triplicate.

### Murine infection model

The tested strains were grown in TSB to exponential growth phase (OD_600_ ≈1.0). After washing with PBS twice, the bacteria was adjusted to different concentration in PBS and administered into eight female Balb/c mice (6 week-old) mice via retro-orbital injection.

For survival curve, the mice were injected with 100 μl bacterial suspension (~1 × 10^8^ CFU) and observed for 4 days. The survival was compared by Log-rank (Mantel-Cox) test with Prism.

To analyze the bacterial loads, the bacterial suspension was adjusted to ~0.5 × 10^7^ CFU. At day 3 post infection, all mice were euthanized, and both kidneys and livers were harvested and ground for bacterial CFU counting on tryptic soy agar. The plates were incubated at 37°C overnight and colonies were enumerated.

### Statistical analysis

All the statistical analysis was performed by unpaired, two-tailed Student’s *t*-test (Prism version 7.0).

## Results

### Disruption of *clpP* decreases the expression of SaeS in *S. aureus* Newman

**Figure 1.**
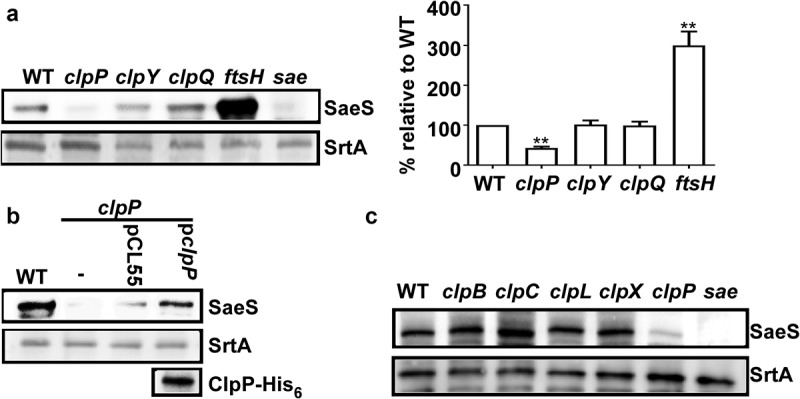
Disruption of *clpP* decreases the expression of SaeS in *S. aureus* Newman. (a) The effect of the mutation in ATP-dependent protease genes on the expression of SaeS. Bacteria were grown in TSB and collected at the stationary growth phase (OD_600_ = 2), then the expression of SaeS was determined by western blot analysis. in this assay, the wild-type (WT), *clpP, clpY, clpQ, ftsH* mutant strains were used. *sae* mutant strain was used as a negative control. data were derived from three biological repeats. Sortase (SrtA) was used as a loading control. **, p < 0.01 (versus WT) by unpaired, two-tailed student’s *t*-test. the quantification of the western blot results is shown on the right. (b) Complementation analysis of the ClpP-mediated regulation of SaeS. Cells were grown as above. SaeS was detected by western blot analysis. the ClpP protein for the complemented strain was determined by His antibody. pCL55, the vector control; p*clpP*, pCL55 carrying the *clpP* gene. (c) The effect of the mutation in ClpATPases genes on the expression of SaeS. As with above, SaeS was detected by western blot analysis.The original blots were presented in Supplementary Figure S3, and cropping lines are indicated in red color.

Previously, we showed that SaeS L18P, the SaeS variant in the Newman strain, is degraded by the ATP-dependent protease FtsH [10]. To examine the role of other ATP-dependent proteases in the regulation of SaeS L18P, we carried out a Western blot assay for SaeS in the protease mutants of *S. aureus* Newman. As expected, the deletion of *ftsH* increased the protein level of SaeS (Figure 1(a)). Intriguingly, when *clpP* was deleted, almost no SaeS was detected, whereas the mutations in either *clpY* or *clpQ* did not significantly affect the SaeS expression (Figure 1(a)). Subsequent complementation with a plasmid carrying the *clpP* gene further confirmed the positive role of ClpP in SaeS production (Figure 1(b)). When ClpATPase genes were disrupted, no significant changes were observed in the expression level of SaeS (Figure 1(c)), indicating that either the Clp ATPases are not involved in the regulation of SaeS expression, or they have redundant roles.

### The *clpP* mutation reduces the overall *sae* activity

**Figure 2.**
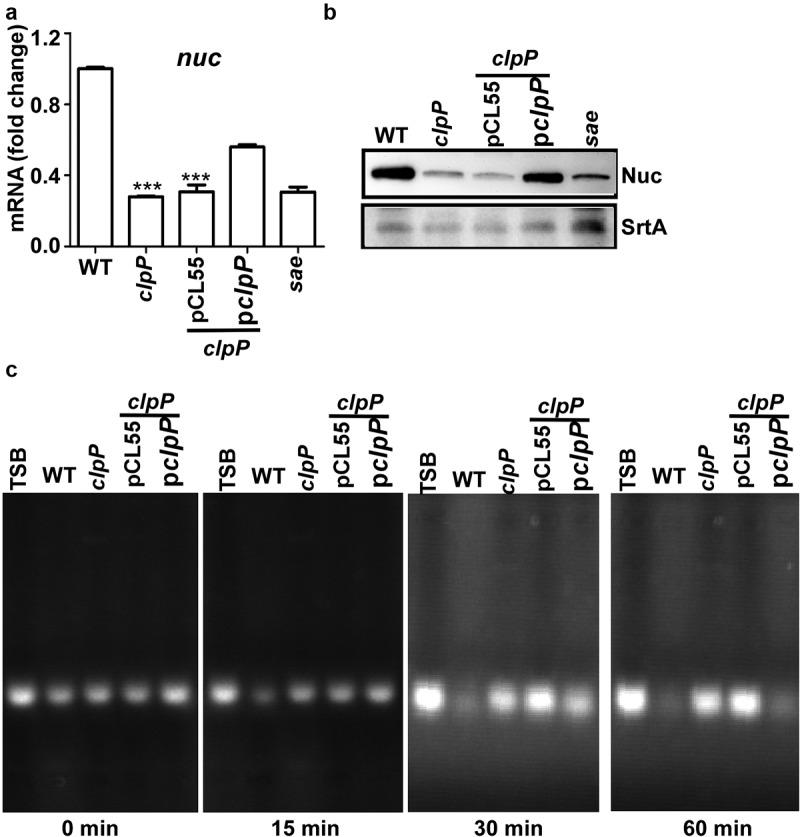
The ClpP-mediated regulation of SaeS affects the production of nuclease. (a) The effect of *clpP* mutation on the transcription of *nuc*, the gene encoding nuclease. Cells were grown in TSB until the stationary growth phase (Od_600_ = 2). The transcript level of *nuc* was measured by RT-PCR. Data were derived from three biological repeats. *sae* mutant strain was used as a negative control. ***, p < 0.001 (versus WT) by unpaired, two-tailed Student’s *t*-test. (b) The effect of *clpP* mutation on the expression of Nuc protein. Cells were prepared as above. The original figure was presented in Supplementary Figure S4, and cropping lines are indicated in red color. SrtA was used as a loading control. (c) The effect of *clpP* mutation on the nuclease activity of culture supernatant. Sterile TSB or culture supernatants were incubated at 37°C with salmon sperm DNA. at 30 min, samples were withdrawn and subjected to agarose gel electrophoresis followed by staining with ethidium bromide. WT, wildtype; *clpP*, *clpP* mutant; pCL55, a vector control; p*clpP*, pCL55 containing the *clpP* gene. *sae*, *sae* mutant; TSB, TSB medium.

The SaeRS TCS is essential for producing more than 20 staphylococcal virulence factors, including nuclease (Nuc) [6,12]. To assess whether the reduced level of SaeS in the *clpP* mutant affected the overall activity of the SaeRS TCS, we measured the transcript level of the *nuc* gene as an indicator of the Sae activity in the WT and the *clpP* mutant of the Newman strain. As shown, the transcript level of *nuc* was significantly reduced in the *clpP* mutant, and it was partially restored by a complementation plasmid carrying the *clpP* gene (Figure 2(a)). The reason for the partial complementation is not clear. Since the complementation plasmid is integrated in the *geh* gene, the expression of the *clpP* gene in the plasmid might be affected by genetic elements (e.g. *geh* promoter) of nearby region. Nonetheless, the transcript analysis results were mirrored by Western blot and nuclease assay: The Nuc expression level and the nuclease activity were reduced by the *clpP* mutation and restored by the *clpP* complementation plasmid (Figure 2(b,c)). Based on these results, we concluded that the ClpP-mediated regulation of SaeS is required for the full activity of the SaeRS TCS in the strain Newman.

### ClpP can affect the SaeS L18P expression in both an FtsH-dependent and independent manner

**Figure 3.**
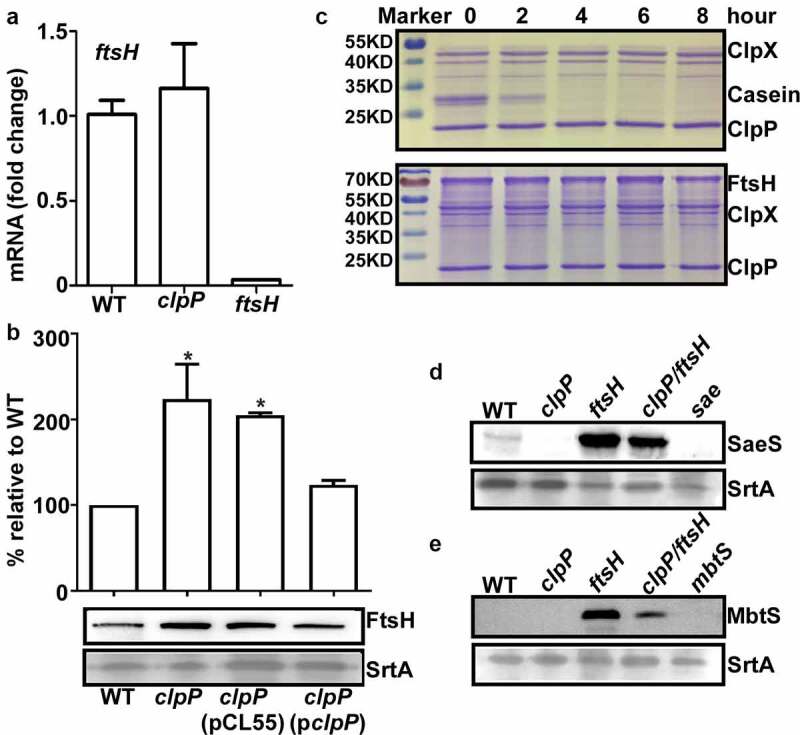
ClpP can affect the SaeS L18P expression in both an FtsH-dependent and independent manner. The transcription of the *ftsH gen*e (a) and the expression of FtsH (b) were determined in samples prepared from stationary-phase (OD_600_ = 2) cells grown in TSB. *, p < 0.05 (versus WT) by unpaired, two-tailed Student’s *t*-test. The quantification of the Western blot results is shown on the right. (c) FtsH is not a substrate of ClpP *in vitro*. Purified 6-His tag ClpX, ClpP, FtsHc by Ni-NTA agarose were determined by the bicinchoninic acid (BCA) assay and used in the protease degradation assay *in vitro*. The substrates α-casein and FtsHc were added to the reaction mix independently. Samples were collected at the given point of time, 15 μl samples were taken, and added 2 × SDS loading buffer to stop the reaction. Results were presented by SDS-PAGE with coomassie blue staining. Cells were grown in TSB until the stationary growth phase (Od_600_ = 2); then, the levels of the SaeS (d) and MbtS (e) protein were measured by Western blot analysis. WT, wild-type; *clpP*, the *clpP* mutant; *ftsH*, the *ftsH* mutant; *clpP/ftsH*, the *clpP*/*ftsH* double mutant; *sae* and *mbtS* mutant strains were used as negative control. SrtA was used as a loading control. Full-length blots are presented in Supplementary Figure S4, and cropping lines are indicated in red color.

Since FtsH degrades SaeS L18P in Newman strain [10], ClpP might exert its positive effect on the SaeS L18P expression via FtsH. Indeed, although the transcription of *ftsH* was not affected by ClpP (Figure 3(a)), the protein level of FtsH was slightly increased in *clpP* mutant strain and restored to the WT level by the complementation plasmid (Figure 3(b)). These results indicate that ClpP has a destabilization effect on FtsH, which can contribute to the increase of the SaeS L18P expression. In an *in vitro* proteolysis assay, however, ClpXP did not degrade FtsH at least until 8 hr, whereas it did degrade α-casein within 2 hrs (Figure 3(c)). Therefore, it appears that ClpP destabilizes FtsH indirectly or utilizes other chaperones than ClpX.

If ClpP positively controls the SaeS L18P expression only via FtsH, the expression level of SaeS L18P in *ftsH* mutant would not be affected by further mutation in *clpP*. To our surprise, however, the SaeS level in *clpP/ftsH* double mutant was significantly lower thanthatin *ftsH* mutant (Figure 3(d)). When we analyzed the expression level of MbtS, another known FtsH substrate, in *ftsH* and *ftsH*/*clpP* mutants [25], similar results were obtained: the mutation of *clpP* in *ftsH* mutant greatly reduced the expression level of MbtS (*ftsH* vs. *clpP/ftsH* in Figure 3(e)), demonstrating that ClpP can exert a positive regulatory effect on FtsH substrates in an FtsH-independent manner.

### MoeA suppresses the hemolytic activity of *S. aureus*

**Figure 4.**
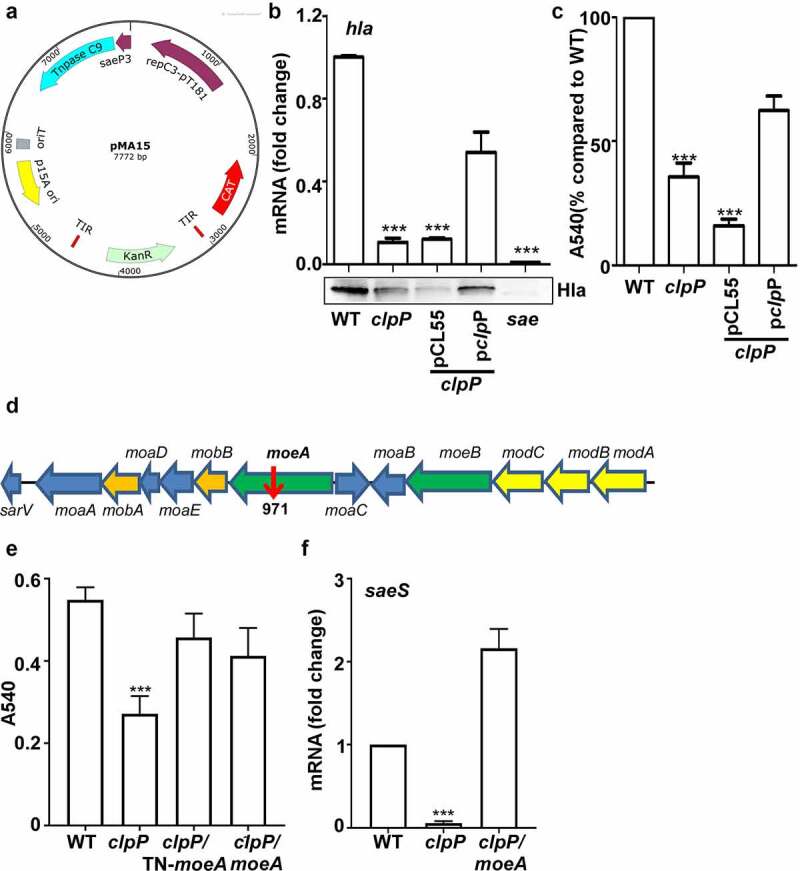
MoeA suppresses the hemolytic activity of *S. aureus*. (a) The plasmid map for pMA15. (b) The effect of *clpP* mutation on the expression of alpha-hemolysin. Cells were grown until the stationary growth phase (OD_600_ = 2). the levels of *hla* mRNA were measured by quantitative RT-PCR. Full-length blots are presented in Supplementary Figure S4 and cropping lines are indicated in red color. (c) The effect of *clpP* mutation on the hemolysis activity of culture supernatant. (d) Genes involved in the synthesis and regulation of MoCo in *S. aureus*.The red arrow is the transposon insertion site. (e) The effect of transposon and deletion mutant on the hemolysis activity of culture supernatant. Cells were grown in TSB until the stationary growth phase (OD_600_ = 2). the filtrates of the bacterial cultures were used to measure the hemolysis activity against human red blood cells. (f) The effect of *clpP/moea* double mutant on the expression of *saeS*. Cells were grown as above. the levels of *saeS* mRNA were measured by quantitative RT-PCR. WT, wildtype; *clpP*, *clpP* mutant; pCL55, a vector control; p*clpP*, pCL55 containing the *clpP* gene. *sae*, *sae* mutant; *clpP/*TN-*moeA*, *moeA* transposon mutant, and *clpP* mutant strain; *clpP/moeA*, *moeA* and *clpP* double mutant. the experiment was carried out in triplicates. ***, p < 0.001 (versus WT) by unpaired, two-tailed Student’s *t*-test.

Next, we decided to identify the bacterial factor mediating the positive effect of ClpP on the SaeS L18P expression. Since ClpP is a protease, we hypothesized that ClpP positively controls SaeS L18P expression by degrading a negative regulatory protein of the SaeS L18P expression and searched for such regulatory protein by transposon mutagenesis with pMA15 (Figure 4(a)). pMA15 is a plasmid containing a temperature-sensitive replicon and a mariner transposon with a kanamycin resistance gene. Since the *clpP* mutation reduces the expression of alpha-hemolysin (*hla*), a known Sae target, and hemolytic activity of culture supernatant (Figure 4(b,c)), we used the hemolytic activity of culture supernatant as an indirect indicator for SaeS L18P expression. We generated transposon insertion mutants in the *clpP* mutant and grew them in a 96-well plate with TSB overnight. Then we collected the culture supernatant for its hemolytic activity. Of 10,000 mutants screened, one (54-2F) showed increased hemolytic activity. Inverse PCR analysis showed that the transposon was inserted in *moeA*, encoding molybdopterin molybdenum transferase (Figure 4(d)). To further confirm that the disruption of *moeA* is responsible for the elevated hemolytic activity of the culture supernatant, we deleted *moeA* in the *clpP* mutant and compared the hemolytic activity of the culture supernatant of the double mutants with that of the *clpP* mutant. As with the transposon mutation, the deletion mutation also increased the culture supernatant hemolytic activity of the *clpP* mutant (Figure 4(e)), confirming the negative regulatory role of the *moeA* gene in the hemolytic activity of *S. aureus*. We hypothesized that the reverse of hemolytic activity may be due to the affection of Sae activity, so the transcription of *saeS* was tested. Before collecting samples for the following experiment, the growth of the tested strains were compared first. We observed that *moeA* deletion mutant did not affect the bacterial growth in TSB, while the *clpP* mutant grew more slowly. Although it was observed only in the early stationary growth phase, the *moeA* mutation restored, at least in partial, the growth of the *clpP* mutant (blue vs. red in the growth curve) (Supplementary Figure S1). All the strains reached the stationary growth phase when OD_600_ was around 2, so the samples were collected at the stationary growth phase. We observed that the transcriptional expression of *saeS* was significantly decreased in *clpP* mutant strain, which can be reversed in *clpP*/*moeA* double mutant strain (Figure 4(f)), suggesting that MoeA may involve in regulating Sae activity.

### MoeA is a ClpXP substrate and a negative regulator of the SaeRS TCS

**Figure 5.**
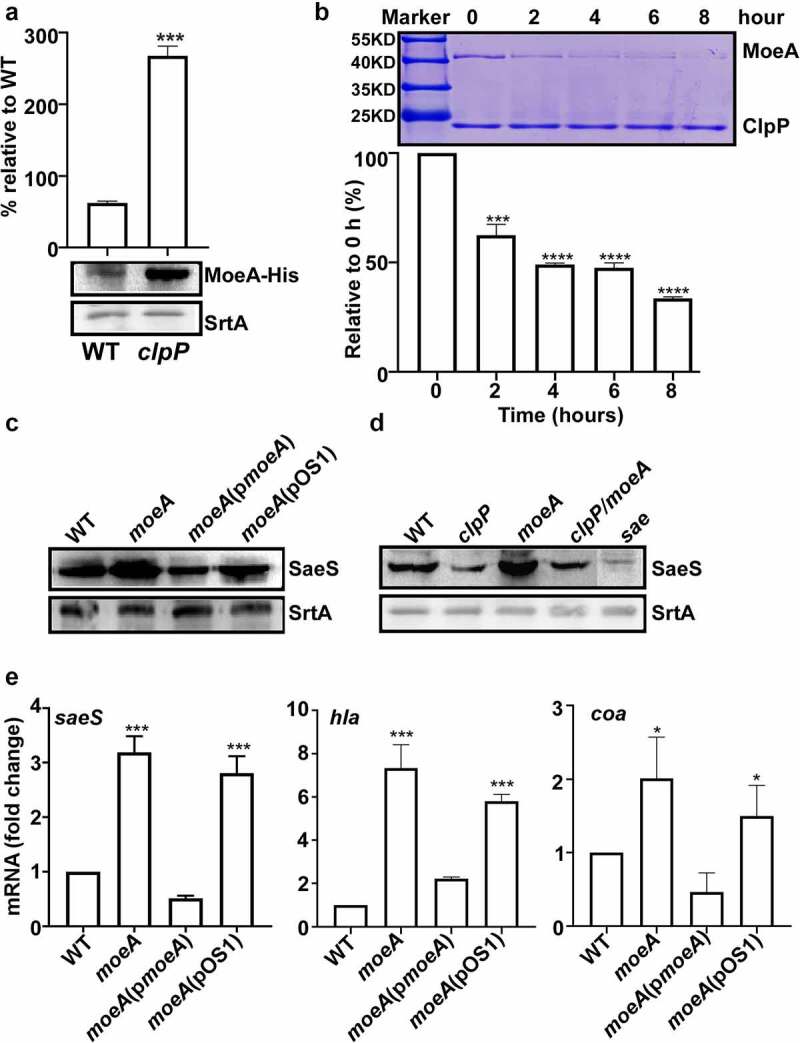
MoeA is a ClpXP substrate and a negative regulator of the SaeRS TCS. (a) Cells were grown in TSB until until the stationary growth phase (OD_600_ = 2); then, the levels of the MoeA protein were measured byWestern blot analysis. ***, p < 0.001 (versus WT) by unpaired, two-tailed Student’s *t*-test. (b) ClpP degrades MoeA *in vitro*. Purified 6-His tag ClpX, ClpP, MoeA by Ni-NTA agarose were determined by the bicinchoninic acid (BCA) assay and used in the protease degradation assay *in vitro*. The substrates MoeA were added in the reaction mix independently. Samples were collected at the given point of time, 15 μl samples were taken and added 2 × SDS loading buffer to stop the reaction. Results were presented by SDS-PAGE with coomassie blue staining. the quantification of the bands is shown at the bottom. ***, p < 0.001 (versus 0 h) by unpaired, two-tailed Student’s *t*-test. (c,d) Cells were grown in TSB until until the stationary growth phase (OD_600_ = 2); then, the levels of the SaeS protein were measured byWestern blot analysis.**, p < 0.01; ***, p < 0.001 (versus WT) by unpaired, two-tailed Student’s *t*-test. (e) Cells were grown as above; then, the levels of the *saeS*, *hla*, and *nuc* transcripts were measured by RT-PCR. ***, p < 0.001 (versus WT) by unpaired, two-tailed Student’s t-test. WT, wild-type; *clpP*, the *clpP* mutant; *moeA*, the *moeA* mutant; pOS1, the vector control; p*moeA*, pOS1 carrying the *moeA* gene; *clpP/moeA*, *moeA* and *clpP* double mutant. SrtA was used as a loading control. Full-length blots are presented in Supplementary Figure S5, and cropping lines are indicated in red color.

If MoeA mediates the ClpP effect on SaeS L18P, MoeA is expected to be regulated by ClpP. Indeed, the expression level of MoeA was significantly higher in the *clpP* mutant, as compared with that in WT (Figure 5(a)). More importantly, MoeA was directly degraded by ClpXP in the *in vitro* condition (Figure 5(b)), demonstrating that MoeA is a substrate of ClpXP.

Since MoeA appears to affect the ClpP regulation on SaeS, we further investigated the role of MoeA in SaeS expression. As shown, the expression of SaeS L18P was significantly increased by the *moeA* deletion, and a plasmid carrying the *moeA* gene restored the SaeS expression to the level of wild type (Figure 5(c)), indicating that MoeA is indeed a negative regulator of the SaeS L18P expression. The elevated SaeS L18P expression in the *moeA* mutant was almost completely abolished to WT level when *clpP* was disrupted (Figure 5(d)). Finally, the transcription of *saeS* and the Sae-target genes, *hla*, and *coa*,was also significantly increased by the *moeA* deletion, which could be restored to the WT level by the *moeA* complementation plasmid (p*moeA* in Figure 5(e)), confirming that *moeA* is a negative regulator for SaeRS TCS.

### MoeA contributes to *S. aureus* pathogenesis in strain Newman

**Figure 6.**
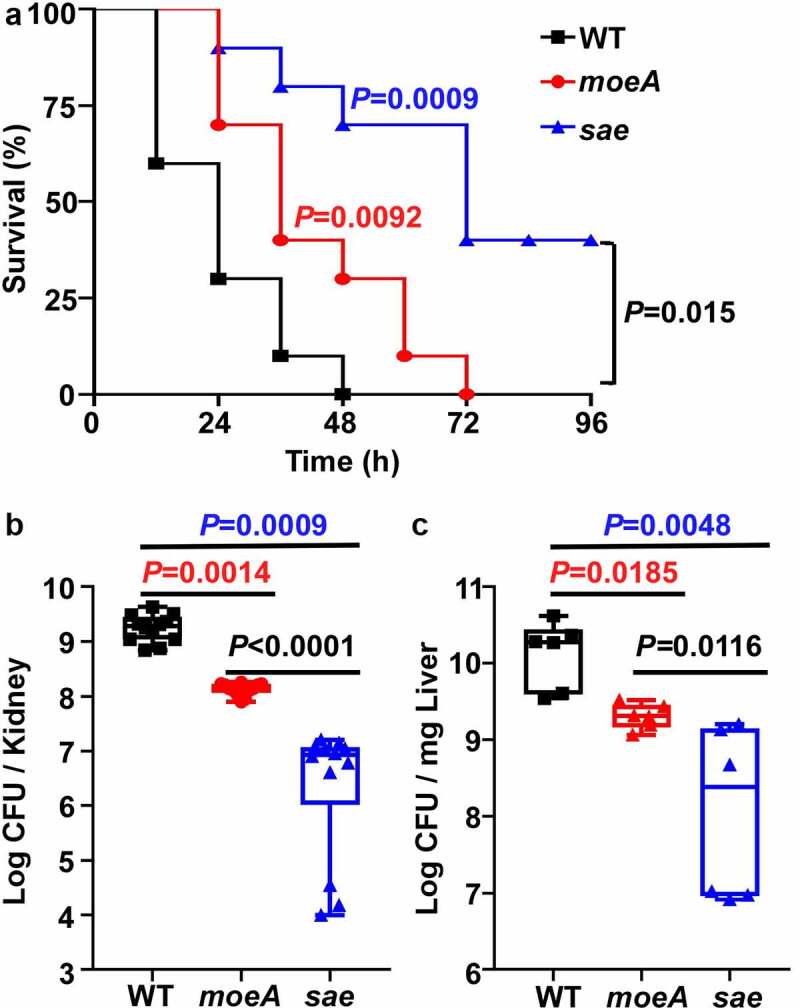
MoeA contributes to *S. aureus* pathogenesis in Strain Newman. (a) The role of MoeA in staphylococcal virulence during bloodstream infection. the test strains were collected at the exponential growth phase. After washing by PBS twice, the test strains were administered to eight mice via retro-orbital route. the significance of the survival was analyzed by Log-rank (Mantel-Cox) test. (b) The role of MoeA in bacteria growth during bloodstream infection. on day 3 post-infection, the mice were killed, and the kidneys were harvested. the samples were ground and diluted serially. the colony-forming unit (CFU) of *S. aureus* was measured by plating on tryptic soy agar. the results were representative of the repeated experiment. the statistical analysis was done by unpaired, two-tailed Student’s *t*-test. WT, wild-type; *moeA*, *moeA* mutant; *sae*, *sae* mutant.

The SaeRS TCS is critical for the virulence of *S. aureus*. Although MoeA is a negative regulator of the SaeRS TCS, it is required to synthesize a molybdenum cofactor (MoCo), which is essential for the activity of various metabolic enzymes. Therefore, we decided to examine the role of MoeA in the virulence of *S. aureus* using a murine blood infection model. When the WT, the *moeA* and the *sae* mutant of Newman were administered to mice via retro-orbital injection, the WT strain killed around 70% of mice by day 1 post-infection; while the *moeA* mutant strain killed 30% of mice during the same time. Obviously, the *moeA* mutant strain took significantly longer time to kill all the mice compared with the WT strain. However, the *sae* mutant strain killed only 60% mice by day 4 post-infection (Figure 6(a)). When the bacterial burden was analyzed for the kidney and liver, significantly lower numbers of both the *moeA* and *sae* mutants were identified, as compared with the WT strain (Figure 6(b,c)). Interestingly, we observed that the bacterial burden was significantly lower in *sae* mutant compared with *moeA* mutant (Figure 6(b,c). These results indicate that, MoeA is critical for the virulence and the *in vivo* survival of *S. aureus*. The suppressive effect on the Sae activity by MoeA also contribute to the virulence using bloodstream infection model.

## Discussion

In *S. aureus*, SaeRS TCS is a signaling system required for the expression of many virulence factors [4]. Previously, we reported that the ATP-dependent protease FtsH directly degrades SaeS L18P in Newman strain [10]. This study found another layer of regulation of the SaeRS TCS in Newman: the cytoplasmic protease ClpP acts as a positive regulator for the SaeRS TCS. Although the molecular mechanism remained to be determined, the ClpP’s positive regulation of the SaeRS TCS is through decreasing the level of FtsH and directly degrading MoeA, a critical enzyme in the MoCo synthesis.

Proteolysis is a key process for the turnover and homeostasis of proteins in bacteria. In *S. aureus*, ATP-dependent protease ClpP and FtsH play important roles in stress resistance and virulence regulation of *S. aureus* [26,27]. Although ClpP has been extensively studied, its functional connection to FtsH has not been identified. In our study, FtsH appears to be negatively regulated by ClpP both in Newman and USA300 strains (Figure 3(b) and Supplementary Figure S2). Moreover, the expression of FtsH-substrate SaeS L18P and MbtS was significantly reduced in *clpP*/*ftsH* double mutant strain compared with *ftsH* single mutant strain (Figure 3(d,e)), indicating that ClpP, as well as FtsH, controls the expression levels of those proteins. However, FtsH appears not to be a substrate for ClpXP (Figure 3(c)). Consistent with our data, FtsH was not identified as a ClpP substrate either by 2-D DIGE (two-dimensional difference gel electrophoresis) or by a ClpP trap method [28,29]. Therefore, although we cannot rule out the possibility that ClpP degrades FtsH via other chaperones than ClpX, it is more likely that ClpP indirectly affects the level of FtsH, possibly by degrading a protein required for the stability of FtsH. This hypothesis predicts that, as with *E. coli* FtsH, which forms a complex with HflKC protein [30], *S. aureus* FtsH might form a complex with other proteins and is subjected to proteolysis by other proteases, likely in the membrane. So far, however, no such FtsH-controlling proteins have been reported in *S. aureus* and await for identification.

To explain the positive regulatory role of ClpP in the SaeRS TCS, initially, we hypothesized that ClpP antagonizes the FtsH activity by degrading a protein required for the stability of FtsH. However, when we tested the hypothesis by a transposon mutagenesis approach, we did not find such a protein factor; instead, we identified MoeA as a mediator for ClpP. As a molybdopterin molybdenum transferase, MoeA catalyzes the conjugation of molybdate to molybdopterin, the last step of the MoCo synthesis. MoCo forms the active site of various enzymes involved in bacterial growth, metabolism, virulence etc [31,32]. For example, in *E. coli* and *Mycobacteria tuberculosis*, MoeA is necessary for nitrate reductase activity and anaerobic metabolism [33,34]. Since MoeA is an enzyme, not a regulatory protein, it is likely that MoeA affects the expression of SaeS L18P indirectly. Although the SaeRS TCS is known to be affected by human neutrophil peptides and fatty acid [35–37], MoeA was never identified as the Sae regulator nor a substrate of ClpP. ClpP is also known to act as a global regulator in oxidative stress response and virulence [12]. Therefore, it is possible that some of the ClpP activity might be mediated by its degradation of MoeA and blocking MoCo synthesis. The linkage between the metabolism disturbance by MoeA mutation and the phenotypes of ClpP mutation needs to be further clarified.

This study demonstrated that MoeA not only is directly degraded by ClpXP but also serves as a negative regulator for the SaeRS TCS in the Newman strain (Figures 4and 5) Intriguingly, although the transcription of *saeS* was reversed in *clpP*/*moeA* double mutant strain (Figure 4(f)), SaeS L18P expression was lower in *clpP*/*moeA* double mutant strain than in the *moeA* deletion mutant (Figure 5(d)). It is likely that the increased level of FtsH contributes to SaeS L18P degradation in the *clpP*/*moeA* double mutant strain (Figure 3(b)). Although SaeS L18P expression is still lower in *clpP*/*moeA* double mutant strain compared with WT (Figure 5(d)), both strains showed a similar hemolytic activity (Figure 4(e)). It is known that the SaeS L18P has constitutively high sensor kinase activity, and, as a high-affinity target of the SaeRS TCS, alpha-hemolysin is insensitive to a moderate decrease of the Sae activity [38]. Therefore, it is possible that the lowered Sae activity in the *clpP*/*moeA* double mutant is still sufficient to activate the alpha-hemolysin synthesis and maintain the hemolytic activity.

Although *moeA* is a negative regulator for the virulence regulator SaeRS, its disruption did not increase the virulence of *S. aureus*; on the contrary, the *moeA* mutant showed lower virulence and survival *in vivo* (Figure 6). However, the regulation on SaeRS compensate for the virulence partially, the *moeA* mutant displayed higher virulence compared with the *sae* mutant in the bloodstream infection model (Figure 6). In *S. aureus*, MoCo is a cofactor for nitrate reductase and formate hydrogenase, enzymes for anaerobic nitrate respiration [39]. Therefore, when the nitration respiration is interrupted by the *moeA* mutation, the higher activity of the SaeRS TCS and expression of higher levels of virulence factors seem insufficient to support the bacterial survival. Previously, Mashruwala, et al., reported that the supplementation of nitrate to anaerobic *S. aureus* culture suppressed the SaeRS-dependent biofilm formation and proposed a model where the SaeRS TCS responds to the cellular respiratory status [40]. Therefore, the negative regulatory role of the *moeA* gene in SaeS expression might be due to the disruption of the anaerobic nitrate respiration. Nonetheless, how the SaeRS TCS senses the cellular respiratory status remains unknown and warrants further investigation.

In conclusion, we provided evidence that, in *S. aureus* Newman, ClpP positively affects the expression of SaeS L18P by destabilizing FtsH and degrading the metabolic protein MoeA. In *S. aureus*, the cytoplasmic ClpP proteolytic system appears to be functionally connected to the membrane protease FtsH. The metabolic protein MoeA is also involved in the regulation of SaeS L18P expression by unknown mechanism. The ClpP-mediated degradation of MoeA indicates that MoeA might be the focal regulatory point where ClpP affects metabolism and virulence simultaneously.

## Supplementary Material

Supplemental Material

## Data Availability

The authors confirm that the data supporting the findings of this study are available within the article and its supplementary materials.
