# Supplementary material for: Molybdopterin biosynthesis pathway contributes to the regulation of SaeRS two-component system by ClpP in Staphylococcus aureus
Source: Virulence. 2022 Apr 28;13(1):727–39. doi: 10.1080/21505594.2022.2065961 (PMC9067530; doi:10.1080/21505594.2022.2065961)
Supplement: Supplemental Material [file KVIR_A_2065961_SM4531.docx]

**Supplementary Figure 1. The effect of the *clpP*- and the *moeA*-deletion on the growth of *S. aureus*.** OD_600_ in TSB was measured every hour for 24 h. WT, wildtype; *clpP*, *clpP* mutant; *clpP*(pCL55), a vector control; *clpP*(p*clpP*), pCL55 containing the *clpP* gene; *moeA*, the *moeA* mutant; *moeA*(pOS1), the vector control; *moeA*(p*moeA*), pOS1 carrying the *moeA* gene; *clpP/moeA*, *clpP* and *moeA* double mutant.


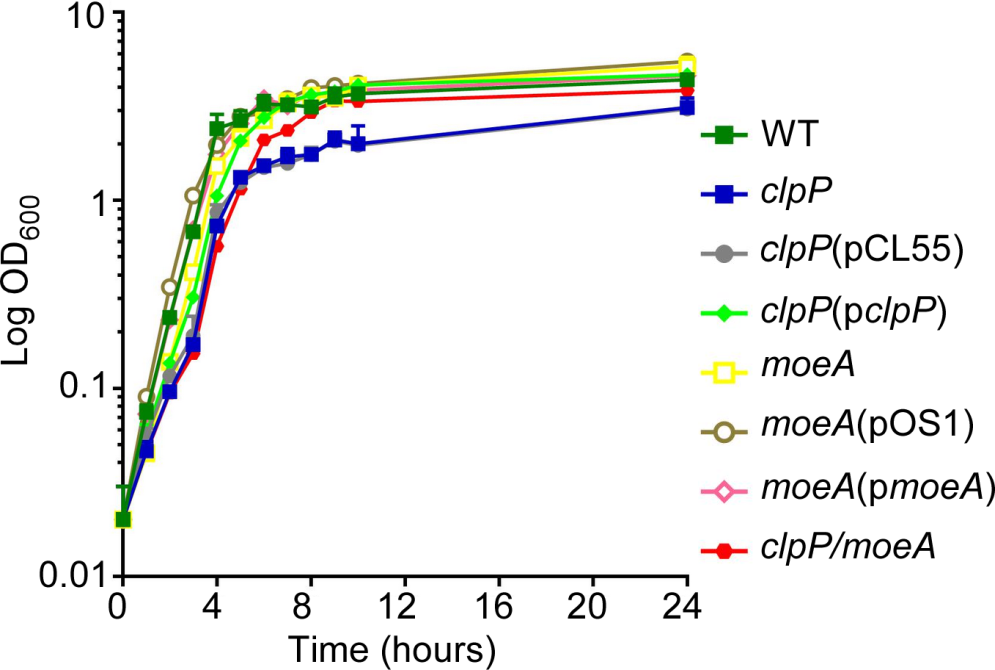


**Supplementary Figure 2. ClpP affect FtsH in USA300.** The expression of FtsH was determined in samples prepared from stationary-phase (OD_600_ = 2) cells grown in TSB using Western blot. SrtA, Sortase A was used as loading control.


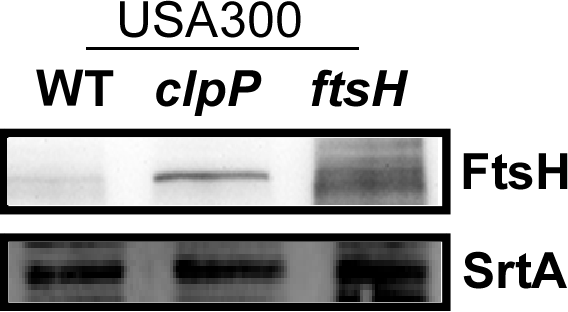


**Supplementary Figure 3.** The original figures for Figure 1.

**
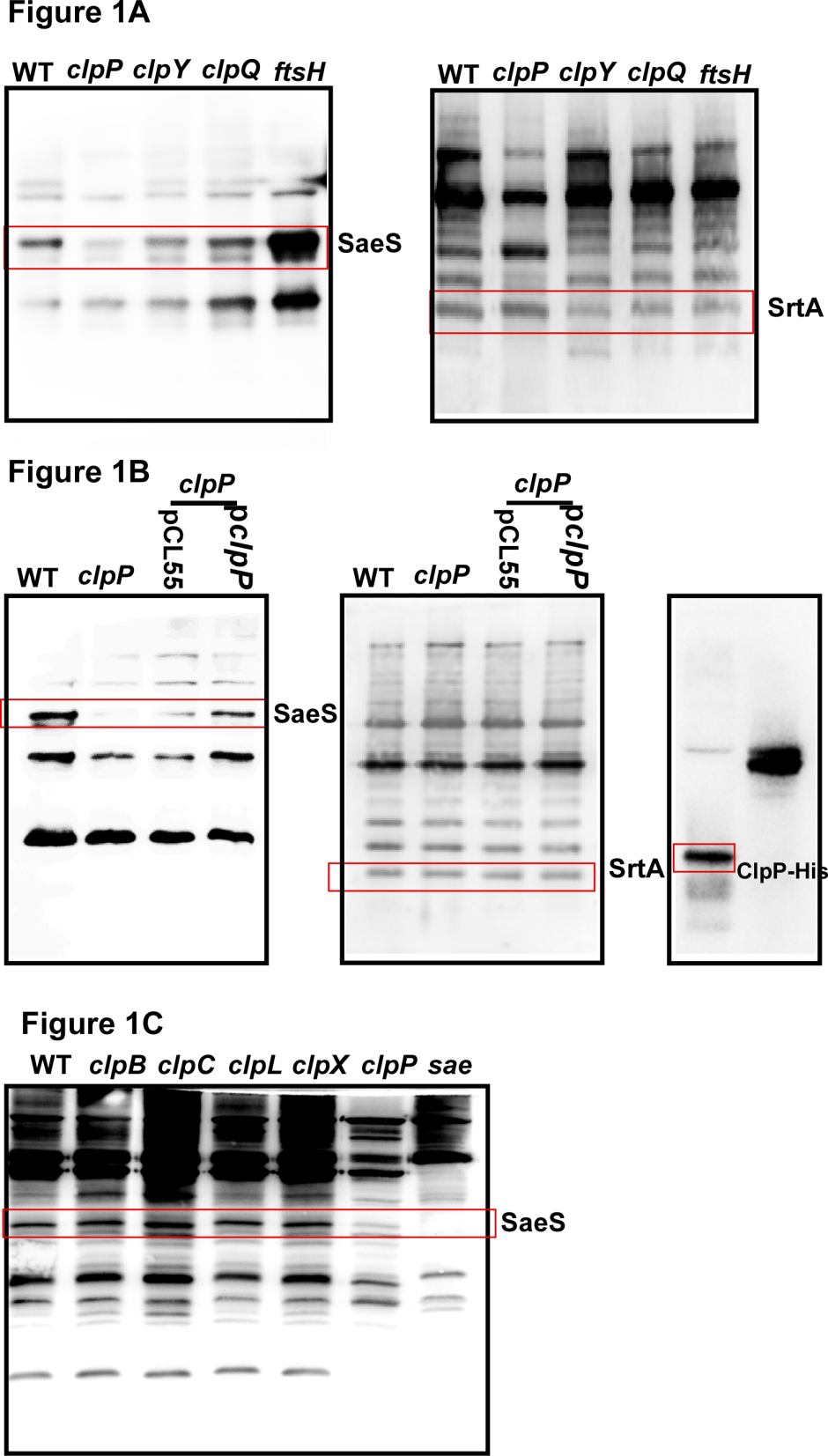
**

**Supplementary Figure 3.** The original figures for Figures 2, 3, 4.


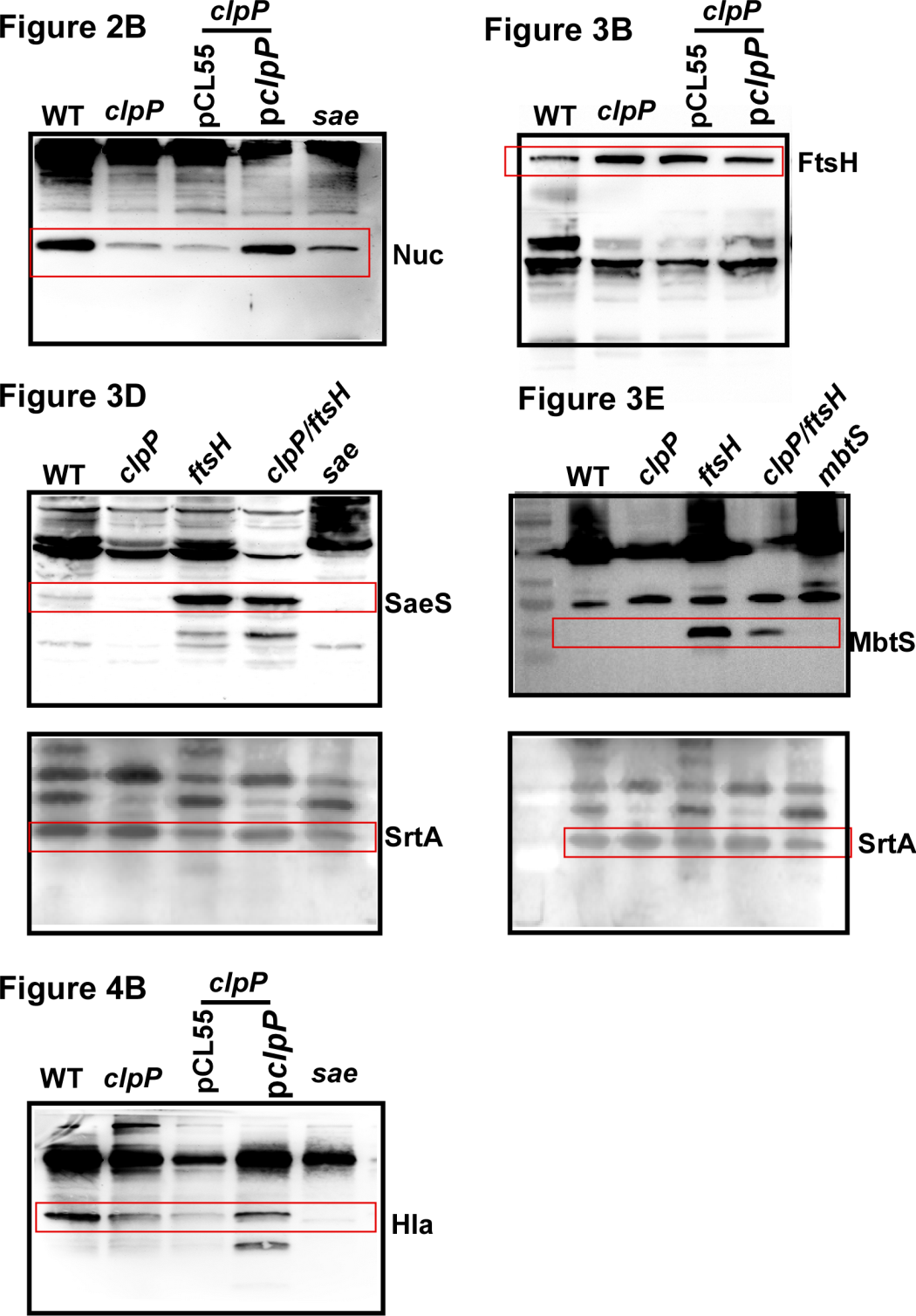


**Supplementary Figure 4.** The original figure for Figure 5.


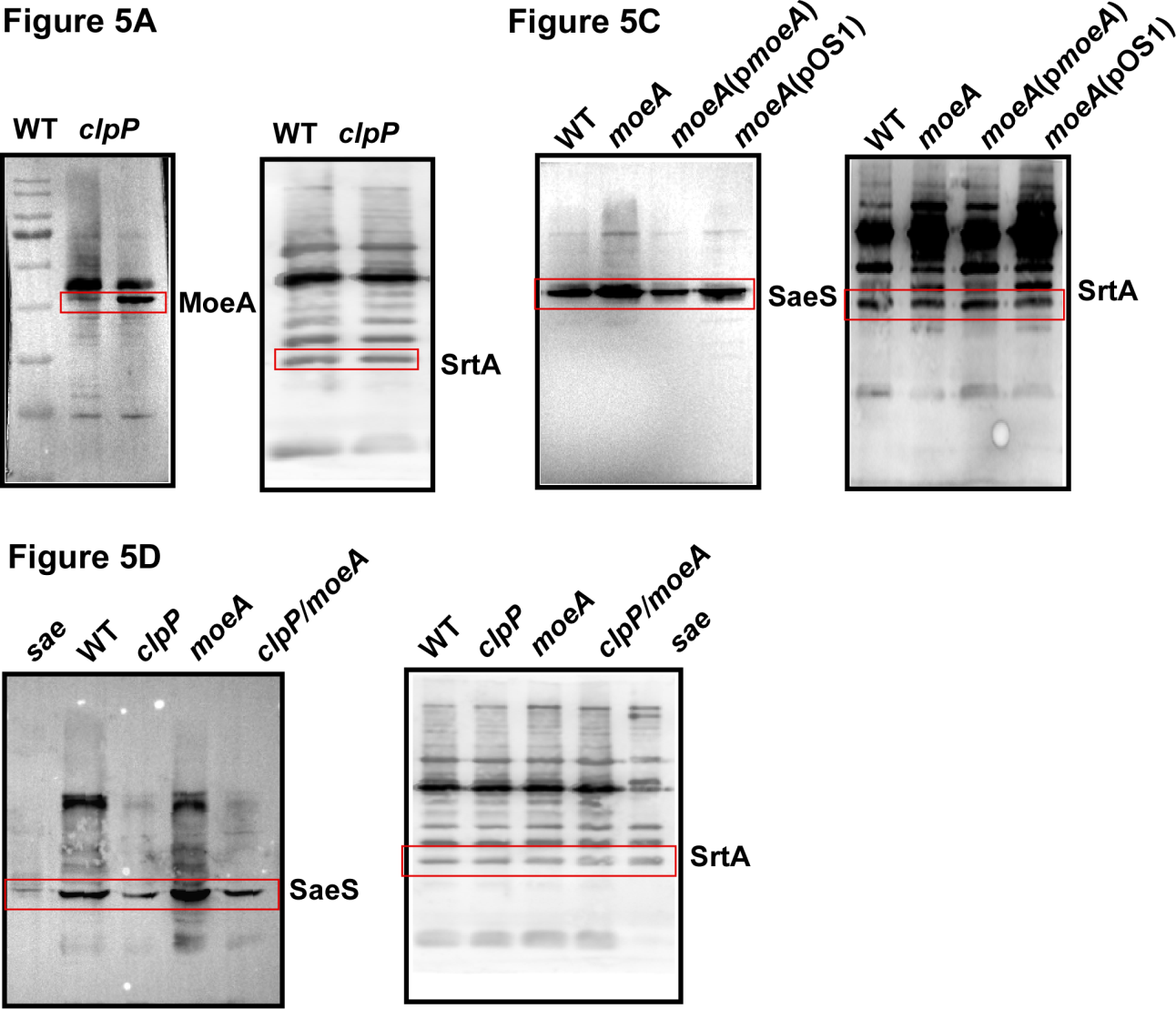


**Supplementary Table 1. Bacterial strains and plasmids used in this study.**

| **Strain or plasmid** | **Relevant characteristic** | **Origin or reference** |
| --- | --- | --- |
| ***E. coli*** |  |  |
| DH5α | Plasmid free, restriction deficient | New England Biolabs |
| ***S. aureus*** |  |  |
| RN4220 | Restriction deficient, prophage cured | [^1^](#_ENREF_1) |
| Newman | Clinical isolate, L18P substitution in SaeS | [^2^](#_ENREF_2) |
| NMΔ*clpQ* | Newman with deletion of the *clpQ* | This study |
| NMΔ*ftsH* | Newman with deletion of the *ftsH* | [^3^](#_ENREF_3) |
| ΦΝΞ-17440 | Transposon mutant of *clpY*, Phoenix library | [^4^](#_ENREF_4) |
| NM*clpP* | Newman strain that acquired the *clpP* transposon mutation in NE912 via transduction | [^3^](#_ENREF_3) |
| NM*clpB* | Newman strain that acquired the *clpB*transposon mutation in NE967 via transduction | [^3^](#_ENREF_3) |
| NM*clpC* | Newman strain that acquired the *clpC* transposon mutation in NE699 via transduction | [^3^](#_ENREF_3) |
| NM*clpL* | Newman strain that acquired the *clpL* transposon mutation in NE1219 via transduction | [^3^](#_ENREF_3) |
| NM*clpX* | Newman strain that acquired the *clpX* transposon mutation using mariner transposon | [^3^](#_ENREF_3) |
| ***Plasmid*** |  |  |
| pKOR1 | Allelic replacement plasmid | [^5^](#_ENREF_5) |
| pKOR1Δ*moeA* | pKOR1 containing*moeA* deletion cassette | This study |
| pIMAY | Allelic replacement plasmid | [^6^](#_ENREF_6) |
| pIMAYΔ*clpQ* | pIMAY containing*clpQ* deletion cassette | This study |
| pCL55 | An integration vector for *S. aureus* | [^7^](#_ENREF_7) |
| p*clpP* | pCL55 carrying the *clpP* gene with His-tag sequence at the C-terminus | [^3^](#_ENREF_3) |
| p*moeA* | pOS1 carrying the *moeA* gene with His-tag sequence at the C-terminus | This study |

**Supplementary Table 2. Oligonucleotides used in this study**

| **Name** | **Sequence (5’ 🡪 3’)** | **Target** |
| --- | --- | --- |
| P1986 | ATTGGATTGGAAGTAC GCTTTGGCAGTTTATTCTTGACATGTA | LIC for pIMAY |
| P1987 | ATTGGAAGTGGATAAC CGAAGTGATCTTCCGTCACAGGTATT | LIC for pIMAY |
| P2391 | TACTTCCAATCCAATG CCGATTAGTTACAATTATTTAATTC | For *clpQ* deletion |
| P2392 | TATTACTCATGTTTCATTCTCCT | For *clpQ* deletion |
| P2393 | AGGAGAATGAAACATGAGTAATA TCAGAGCACGATAAATAATTAC | For *clpQ* deletion |
| P2394 | TTATCCACTTCCAATG GGTTTGAATAACGCTACCTTC | For *clpQ* deletion |
| P236 | ATTGGAAGTGGATAACGGTACCGGTTCCGAGGCTC | LIC for pKOR1 |
| P237 | ATTGGATTGGAAGTACGGGCCCGAGCTTAAGACTGG | LIC for pKOR1 |
| PN1 | TACTTCCAATCCAATGAGTCAATGCTTGTTCAAGCAC | For *moeA* deletion |
| PN2 | TACTTCCAATCCAATGAGTCAATGCTTGTTCAAGCAC | For *moeA* deletion |
| PN3 | AGTTCCTCTTCAGCAGCGTCAGTGCTACCGTAATTGCCGG | For *moeA* deletion |
| PN4 | TTATCCACTTCCAATGCGTACTAACGGTATCAGATACT | For *moeA* deletion |
| PN5 | GAGCCCGGGATGCCTCATCAATGTTGTCT | For *moeA* complement |
| PN6 | GAGGGATCCCTAATGATGATGATGATGATGATGACCCCTCCTAGTGCATCTAGTATAT | For *moeA* complement |
| PN7 | GCGGGATCCGAGTAAACTCAGTCATTTTACCCCV | For *moeA* expression |
| PN8 | GCGGAATTCGCTAAAAGAAGTTCCTCTTCAGCAGCG | For *moeA* expression |
| P392 | CTGATTACTATCCAAGAAATTCGATTG | Real-time PCR for *hla* |
| P393 | CTTTCCAGCCTACTTTTTTATCAGT | Real-time PCR for *hla* |
| P378 | GTCTAAGTAGCTCAGCAAATG | Real-time PCR for *nuc* |
| P379 | GTCATTGGTTGACCTTTGTAC | Real-time PCR for *nuc* |
| P516 | GATGATATGACTCATATTATCAC | Real-time PCR for *saeS* |
| P517 | CGTTACAGAAATTCACTTCTAATG | Real-time PCR for *saeS* |
| P195 | GGTGGCGGTAGTGGCGGTCG | Real-time PCR for *ftsH* |
| P196 | GACACCTTTAGGAATCCTAG | Real-time PCR for *ftsH* |
| P43 | CAAATGATCACAGCATTTGGTACAG | Real-time PCR for *gyrB* |
| P44 | CGGCATCAGTCATAATGACGAT | Real-time PCR for *gyrB* |

**REFERENCES**

1. Kreiswirth BN, Lofdahl S, Betley MJ, O'Reilly M, Schlievert PM, Bergdoll MS, et al. The toxic shock syndrome exotoxin structural gene is not detectably transmitted by a prophage. Nature 1983; 305:709-12.

2. Duthie ES, Lorenz LL. Staphylococcal coagulase; mode of action and antigenicity. Journal of general microbiology 1952; 6:95-107.

3. Liu Q, Wang X, Qin J, Cheng S, Yeo WS, He L, et al. The ATP-Dependent Protease ClpP Inhibits Biofilm Formation by Regulating Agr and Cell Wall Hydrolase Sle1 in *Staphylococcus aureus*. Front Cell Infect Microbiol 2017; 7:181.

4. Bae T, Banger AK, Wallace A, Glass EM, Aslund F, Schneewind O, et al. *Staphylococcus aureus* virulence genes identified by bursa aurealis mutagenesis and nematode killing. Proc Natl Acad Sci U S A 2004; 101:12312-7.

5. Bae T, Schneewind O. Allelic replacement in *Staphylococcus aureus* with inducible counter-selection. Plasmid 2006; 55:58-63.

6. Monk IR, Shah IM, Xu M, Tan MW, Foster TJ. Transforming the Untransformable: Application of Direct Transformation To Manipulate Genetically *Staphylococcus aureus* and *Staphylococcus epidermidis*. MBio 2012; 3.

7. Lee CY, Buranen SL, Ye ZH. Construction of single-copy integration vectors for *Staphylococcus aureus*. Gene 1991; 103:101-5.
